# Preparation of a Near-Infrared Ray Absorption Film from *N*-Phenylthiocarbamoyl Chitosan Derivative

**DOI:** 10.3390/ijms161226153

**Published:** 2015-12-04

**Authors:** Shouko Nishida, Masaya Shibano, Hiroshi Kamitakahara, Toshiyuki Takano

**Affiliations:** Division of Forest and Biomaterials Science, Graduate School of Agriculture, Kyoto University, Kyoto 606-8502, Japan; s.nishida.kyoto.agriculture@gmail.com (S.N.); ma.shibano@gmail.com (M.S.); hkamitan@kais.kyoto-u.ac.jp (H.K.)

**Keywords:** chitosan, copper, decanoylation, near-infrared absorption, phenythiocarbamoyl

## Abstract

We recently observed that the decanoylation of *N*-phenylthiocarbamoyl chitosan (**2**) with a mixture of decanoic anhydride and pyridine at 60 °C for 24 h afforded *N*,*N*-(decanoyl)phenythiocarbamoyl-/2-isothiocynato chitosan decanoate (**3b**) rather than the expected product *N*,*N*-(decanoyl)phenylthiocarbamoyl chitosan decanoate (**3a**). This result suggested that some of the *N*,*N*-(decanoyl)phenylthiocarmbamoyl groups had been converted to isothiocyanate groups during the decanoylation process. The subsequent reaction of compound **3b** with aniline gave *N*,*N*-(decanoyl)phenylthiocarbamoyl/*N*-phenylthiocarbamoyl chitosan decanoate (**4**) in high yield. A solution of compound **4** in CHCl_3_ was then added to a solution of copper decanoate (**5**) in the same solvent, and the resulting mixture was cast onto a glass plate to give a cast film. The film was annealed at 200 °C in an oven to give a greenish film, which showed good near-infrared absorption characteristic in the range of 800–2200 nm.

## 1. Introduction

Chitosan is derived from the *N*-deacetylation of chitin, which is the second most abundant of the known natural biopolymers. Although a strict nomenclature has not yet been defined with respect to the degree of the *N*-deacetylation of chitosan, chitin with the degree of *N*-deacetylation of more than 40% is generally considered to be chitosan. Numerous chitosan derivatives have been reported for a wide range of interesting applications, including foods, cosmetics, and pharmaceuticals [1,2,3]. Despite these advances, considerable research interests are still being focused on the development of new high-value added applications for chitosan and its derivatives. *N*-Substituted thiocarbamoyl chitosan derivatives are one of the important groups of functional chitosan derivatives to have been reported to date. For example, *N*-acetyl-, *N*-fluoresceinyl-, and *N*-phenyl-thiocarbamoyl chitosan derivatives have been reported to behave as antimicrobial materials [4], corrosion inhibitors [5], macromolecular fluorophores [6], and metal adsorbents [7,8].

Near-infrared rays are defined as light in the range of 780–2500 nm. Approximately half of the solar energy emitted from the sun caused by near-infrared rays [9,10]. It is therefore envisaged that materials capable of adsorbing near-infrared rays could be used as heat-ray shielding films to prevent a temperature rise in a room and in a car for housing and car windows during the summer season [10], as well as being used as heat-ray absorption films during the winter season and heat storage fibers for winter clothes. The absorbing materials are also expected as optical filters for a plasma display panel [11] and as near-infrared bio-imaging materials [12]. We previously reported that a powder mixture of *N-*phenylthiocarbamoyl chitosan and copper stearate could be heated at 200–250 °C to afford a near-infrared absorption material [13]. However, our subsequent investigation of the absorption capabilities of this material revealed that they were inadequate. For example, the degree of substitution with *N*-phenylthiocarbamoyl groups (DS_PhNHCS_) of the chitosan derivatives prepared in this way was low (0.33) and the powder mixture could not be converted to a cast film because it was not soluble in common organic solvents. However, we recently reported the development of a synthetic method for preparation of *N-*phenylthiocarbamoyl chitosan with a much higher DS_PhNHCS_ value of 0.85 [11]. Notably, we prepared *N*,*N*-(acyl)phenylthiocarbamoyl chitosan acylate from this material and found that it showed good properties as a film for the adsorption of near-infrared rays.

**Scheme 1:**
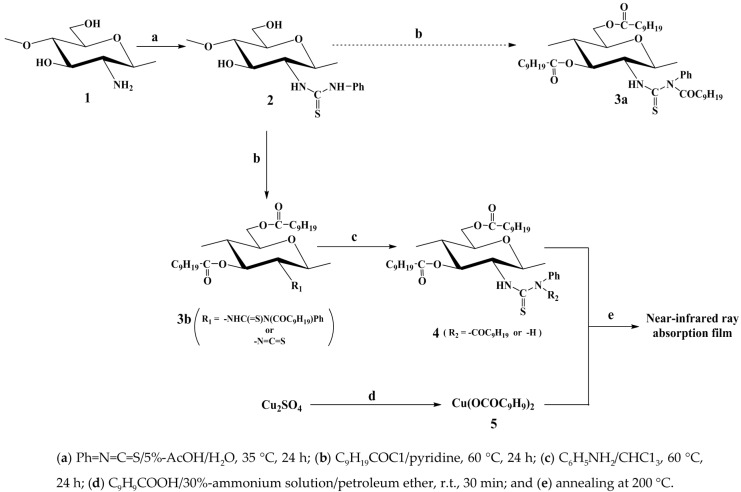
Preparation of a near-infrared ray absorption film.

In this work, *N*,*N*-(decanoyl)phenylthiocarbamoyl chitosan decanoate (**3a**) was initially selected as a promising chitosan derivative for the development of a film for the adsorption of near-infrared rays. This material was selected as a suitable candidate because the results of a preliminary experiment had shown that the introduction of *n*-decanoyl groups at the *O*-3 and *O*-6 positions of chitosan led to an increase in its solubility in several common organic solvents. Surprisingly, however, this process resulted in the preparation of *N*,*N*-(decanoyl)phenylthiocarbamoyl/*N*-phenylthiocarbamoyl chitosan decanoate (**4**) instead of compound **3a** (Scheme 1). This paper describes the preparation of compound **4** from chitosan (**1**) and an evaluation of its behavior as a film for the adsorption of near-infrared rays.

## 2. Results and Discussion

### 2.1. Preparation of N,N-(Decanoyl)phenylthiocarbamoyl Chitosan Decanoate (**4**) 

**Figure 1:**
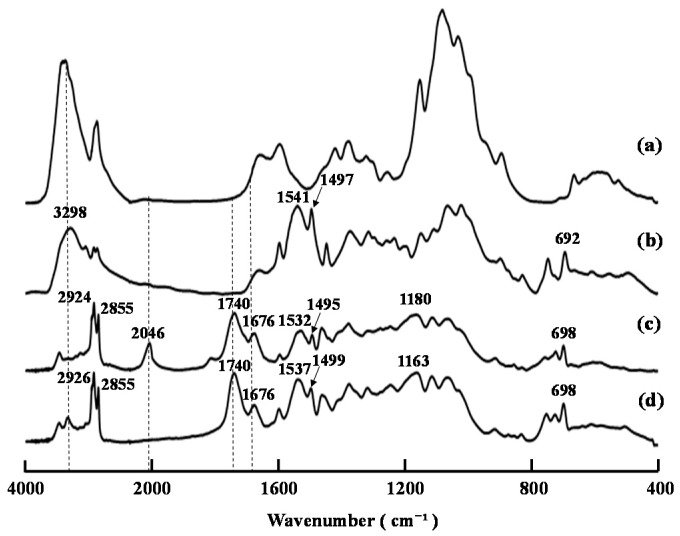
FT-IR spectra of compounds (a) **1**, (b) **2**, (c) **3b** (product (**A**)), and (d) **4**.

The decanoylation of compound **2** [14] with a mixture of decanoic anhydride in pyridine at 60 °C for 24 h afforded product (**A**) in high yield. The FT-IR spectrum of this material contained characteristic ester and amide bands at 1740 and 1676 cm^−1^, respectively, whereas the bands attributed to the hydroxyl groups of the starting material around 3298 cm^−1^ had been reduced significantly (Figure 1). NMR analysis of product (**A**) revealed the characteristic signals of the decanoyl groups at 2.35–0.89 ppm and 33.9–14.1 ppm in its ^1^H and ^13^C NMR spectra, respectively. Taken together, these results suggested that the *O*- and *N*-decanoylation reactions had proceeded smoothly at the *OH*-3, *OH*-6, and *N*-phenylthiocarbamoyl groups of the starting material. Notably, the FT-IR and ^13^C NMR spectra of product (**A**) contained a band at 2046 cm^−1^ and a signal at 141.8 ppm, respectively, which were attributed to the isothiocyanate (NCS) groups [14]. These results suggested that the isothiocyanation reaction had proceeded during the acylation of compound **2** with a mixture of acyl anhydride in pyridine. This result was similar to that reported in our previous paper using an acyl halide/pyridine system [15] and confirmed that product (**A**) was compound **3b** rather than **3a**.

**Figure 2:**
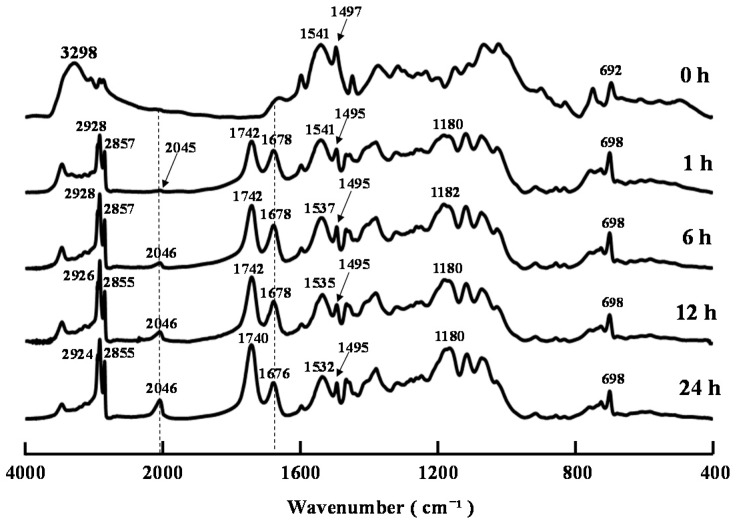
FT-IR spectra of decanoylation products of compound **2** after 0–24 h.

Figure 2 shows the FT-IR spectra of the products resulting from the decanoylation of compound **2** at various time intervals when the reaction temperature was 60 °C. The characteristic bands of the amide groups at 1678 cm^−1^ rapidly appeared within only 1 h, and then gradually decreased, whereas the band belonging to the NCS groups at 2046 cm^−1^ gradually increased with increasing reaction time. This result showed that the formation of the NCS groups was caused by the degradation of the resulting *N,N*-(decanoyl)phenylthiocarbamoyl groups during the *O*-decanoylation. A small band of NCS groups at 2045 cm^−1^ could be detected after only 1 h, suggesting that the formation of NCS groups was unavoidable when the reaction temperature was set as 60 °C.

Wet compound **3b** was reacted with aniline in CHCl_3_ to afford compound **4** in high yield. It is noteworthy that acyl chitosan isothiocyanates became insoluble in several solvents, including tetrahydrofuran (THF), CHCl_3_, and CH_2_Cl_2_ when they are stored in the presence of a drying solid at ambient temperature for more than a week [14]. The characteristic NCS band at 2046 cm^−1^ was found to be absent from the FT-IR spectrum of compound **4**, which suggested that this material was *N*,*N*-(decanoyl) phenylthiocarbamoyl/*N*-phenylthiocarbamoyl chitosan decanoate. The total DS value of *N*,*N*-(decanoyl) phenylthiocarbamoyl and *N*-phenylthiocarbamoyl groups (DS_phenylthiocarbamoyls_) for compound **4** was determined to be 0.82 by elemental analysis. The solubility characteristics of compounds **2** and **4** are summarized in Table 1. Compound **4** was found to be soluble in THF, CHCl_3_, acetone, CH_2_Cl_2_, 1,4-dioxane and DMF, but insoluble in DMSO, MeOH, and H_2_O.

**Table 1:** Solubility of compounds **2** and **4**.

Solvents	THF	CHCl_3_	Acetone	CH_2_Cl_2_	Dioxane	DMF	DMSO	CH_3_OH	H_2_O
δ	9.1	9.3	9.4	9.6	9.8	11.5	12.8	12.9	21.0
Compound **2**	x	x	x	x	x	O	O	x	x
Compound **4**	O	O	O	O	O	O	x	x	x

δ: Solubility parameter; O: Soluble; x: Insoluble.

### 2.2. Preparation of Copper(II) Decanoate (**5**)

**Figure 3:**
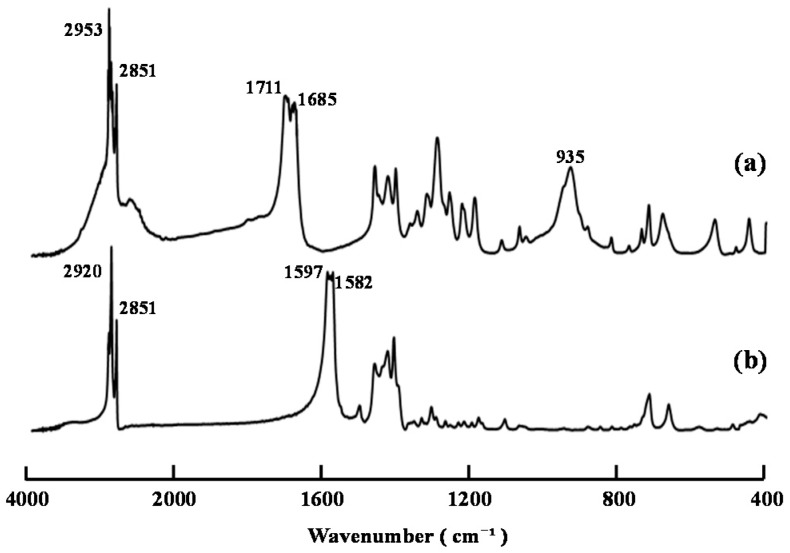
FT-IR spectra of (a) decanoic acid and (b) compound **5**.

Compound **5** was prepared from decanoic acid and copper sulfate according to a modified version of a reported method for the preparation of copper stearate [16]. Figure 3 shows the FT-IR spectra of decanoic acid and compound **5**. The characteristic bands of the carboxyl groups in decanoic acid at 1711 and 1685 cm^−1^ clearly shifted to 1597 and 1582 cm^−1^, and the band of the hydroxyl groups at 935 cm^−1^ disappeared in the FT-IR spectrum of compound **5**. The results were now consistent with the presence of carboxylate groups, which suggested that copper(II) decanoate had been successfully formed. Compound **5** was found to be readily soluble in THF, CHCl_3_, and CH_2_Cl_2_.

### 2.3. Preparation and Characterization of a Cast Film from the Chitosan Derivative **4** and Copper Decanoate (**5**)

**Figure 4:**
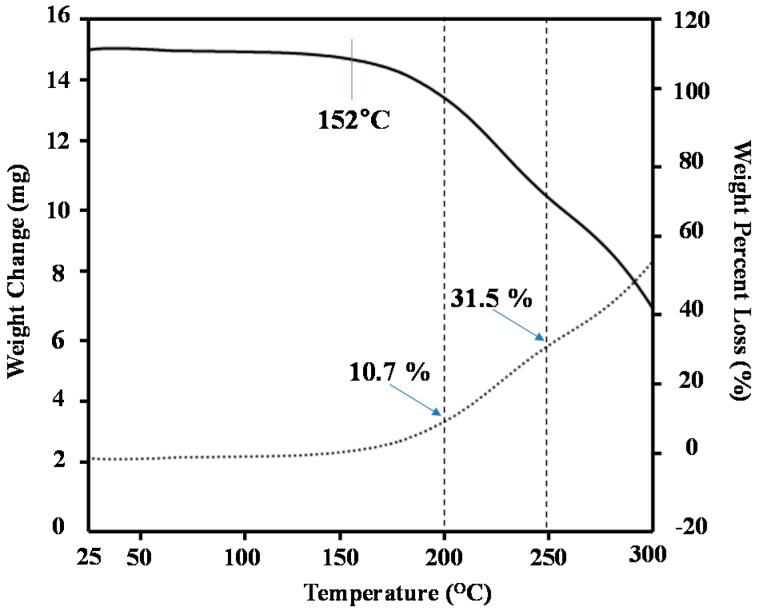
TGA of powder mixture of compounds **4** and **5** with a molar ratio = 1:1.

It has been reported that an annealing temperature of 200–250 °C is essential to allow for the formation of materials with near-infrared ray absorption capabilities from a powdered mixture of *N*-phenylthiocarbamoyl chitosan and copper stearate [13]. With this in mind, we investigated the optimum conditions required to achieve the formation of materials with good near-infrared ray absorption capabilities. We initially subjected a 1:1 (mol/mol) mixture of compounds **4** and **5** to thermogravimetric analysis (TGA) to determine the annealing temperature. The results of this analysis revealed that the thermal degradation of this mixture began at 152 °C, and proceeded smoothly thereafter (Figure 4). The weight percent losses at 200 and 250 °C were 10.7% and 31.5%, respectively. Based on this result, the optimum annealing temperature was determined to be 200 °C.

**Figure 5:**
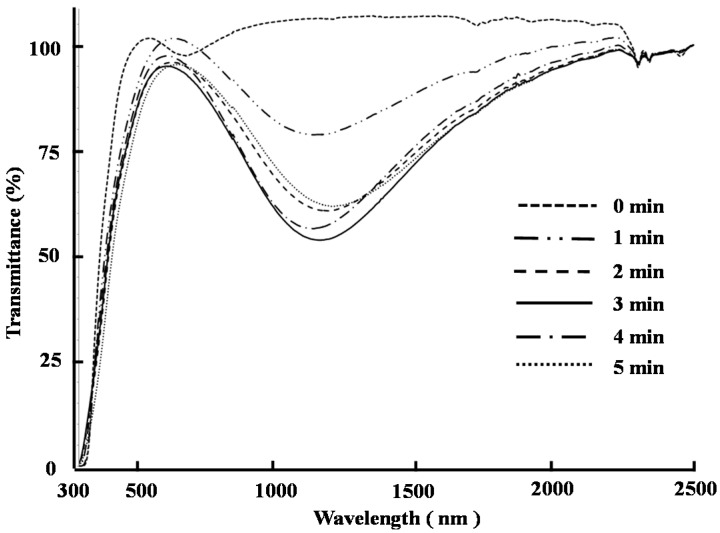
UV-visible-NIR transmittance spectra of films of compounds **4** and **5** with a molar ratio = 1:1 after the heating treatment at 200 °C for 0, 1, 2, 3, 4, and 5 min.

Cast films were subsequently constructed from a 1:1 (mol/mol) mixture of compounds **4** and **5** on glass substrates and annealed at 200 °C for different times in an oven. Figure 5 shows the UV-visible-NIR transmittance spectra of the films treated for different annealing times. The transmittance of light through the films in the range of 800–2200 nm was found to be 100% before the annealing process, which showed that these films possessed no near-infrared ray absorption capability. The transmittance of light in the near-infrared region through the films decreased after an annealing time of 1 min (Figure 5), which indicated that the near-infrared ray absorption ability of the films was increasing with increased annealing time. The level of transmittance in the near-infrared ray region decreased again as the annealing time was increased from 1 to 3 min, but increased only slightly when the annealing time was increased from 3 to 5 min. The transmittance of the film after an annealing time of 3 min was 56.9% at 1166 nm (at the minimum transmittance wavelength). Based on these results, the optimum annealing time was determined to be 3 min.

**Figure 6:**
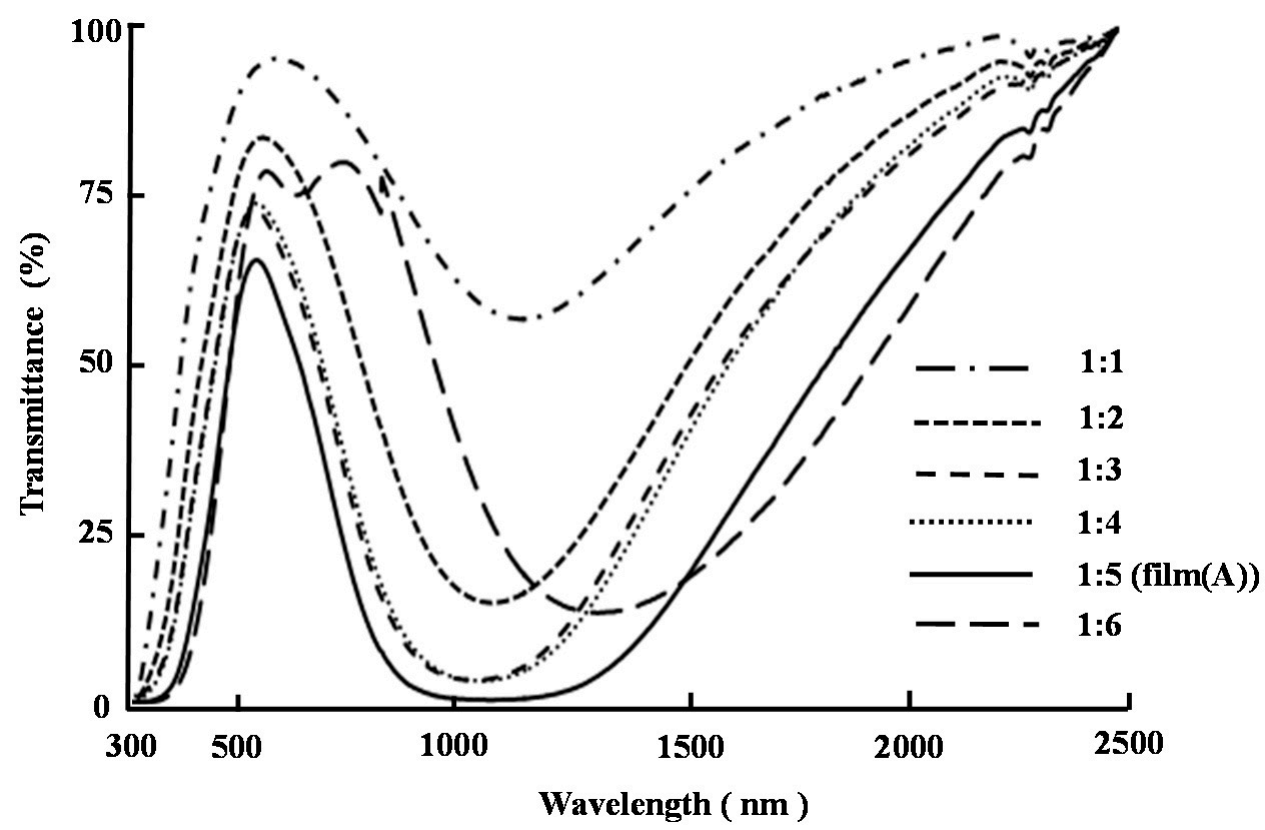
UV-visible-NIR transmittance spectra of films with the molar ratio of compounds **4** and **5** = 1:1, 1:2, 1:3, 1:4, 1:5, and 1:6 after heating treatment at 200 °C for 3 min.

We then investigated the constructed of a variety of different films using different molar ratios of compounds **4** and **5**, which were prepared and treated at 200 °C for 3 min in an oven. Figure 6 shows the UV-visible-NIR transmittance spectra of these films after they had been subjected to the optimal annealing conditions described above. These results revealed that the level of transmittance in the near-infrared region decreased as the ratio of compounds **4** and **5** increased from 1:1 to 1:5, but then increased when the molar ration was increased from 1:5 to 1:6. The reason for this red-shift in the minimum transmittance wavelength of the film with a molar ratio of 1:6 is not yet clear. Based on these results, the optimum molar ratio of compounds **4** to **5** was determined to be 1:5.

**Figure 7:**
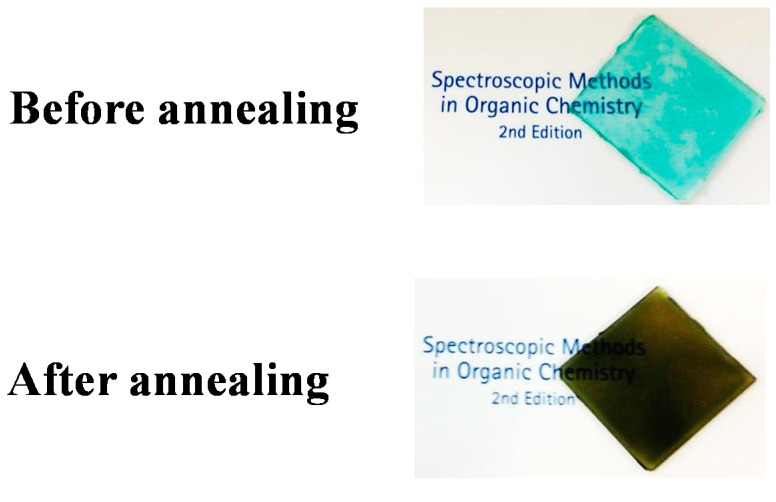
Photograph of film (**A**) (compounds **4**:**5** = 1:5) before and after annealing.

Taken together, the results of the optimization experiments described above showed that films prepared from a 1:5 (mol/mol) ratio of compounds **4** and **5** after an annealing time of 3 min at 200 °C (*i.e.*, film (**A**)) showed the best near-infrared ray absorption ability (Figure 6). Film (**A**) was found to be greenish in color, as shown in Figure 7. The transmittance of film (**A**) was less than 1% at wavelengths in the range of 978–1216 nm, which suggested that film (**A**) possessed excellent near-infrared absorption capabilities. However, the transmittance characteristics of film (A) at wavelengths in the range of 300–750 nm (at visible ray region) decreased compared with how they were before the annealing process. Indeed, the transmittance at 568 nm (at the maximum transmittance wavelength at visible ray region) was 65.6%.

**Figure 8:**
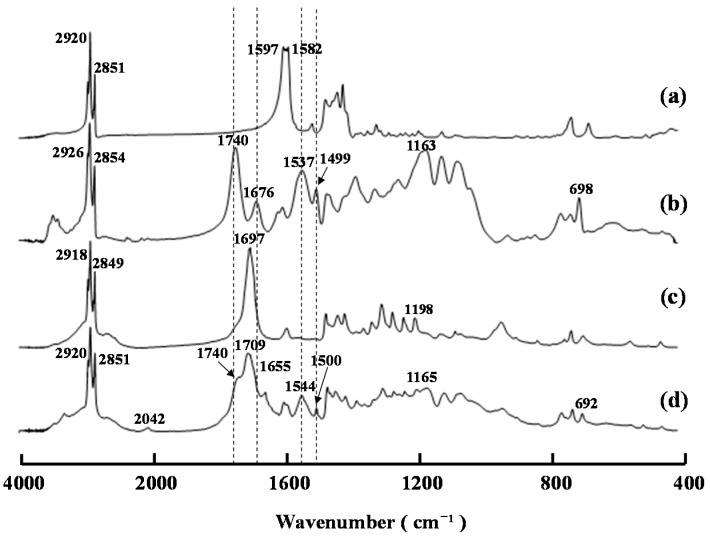
FT-IR spectra of (a) compound **5**; (b) compound **4**; (c) film **A** before the heating treatment; and (d) film **A** after the heating treatment.

One of the possible reasons for the generation of the near-infrared ray absorption capability of film (**A**) could be the formation of micro particles of copper sulfide. It is noteworthy that the UV-visible-NIR transmittance spectrum of film (**A**) was similar to that of a copper sulfide thin film prepared from copper(II) chloride and thiourea using a chemical bath method followed by a period of annealing at 300 °C [17,18]. It has also been reported that copper(II) ions can promote the desulfurization of *N*-phenylthiourea [19]. With this in mind, we evaluated film (**A**) both before and after the annealing process by FE-SEM and FT-IR spectroscopy. Unfortunately, we only observed a small number of micro particles in film (**A**) after the annealing process, and it was therefore not possible for this film to adsorb near-infrared rays. Figure 8 shows the FT-IR spectra of compounds **4** and **5** and film (**A**) before and after the annealing process. The FT-IR spectrum of film (**A**) before the annealing process (Figure 8c) revealed that there had been a significant decrease in the characteristic carboxylate band of compound **5** at 1597 and 1582 cm^−1^, and that the characteristic ester band of compound **4** at 1740 cm^−1^ had shifted to 1697 cm^−1^. This spectrum also showed that the bands derived from the *N*-phenythiocarbamoyl groups of compound **4** at 1676, 1537, and 1499 cm^−1^ [14] had disappeared, even though the molar ratio of compound **4** in film (**A**) was low. It has been reported that 3,6-di-*O*-decanoyl chitosan dithiocarbamate [20] and *N*-phenylthiocarbamoyl chitosan [7] can absorb copper(II) ions with a high level of selectivity. These results therefore suggested that the copper(II) ions had coordinated to the decanoyl and *N*-phenylthiocarbamoyl groups of compound **4** rather than the decanoic acid groups of compound **5** during the preparation of film (**A**) before the annealing process. The ester band at 1740 cm^−1^ and the bands from the *N*-phenythiocarbamoyl groups at 1655, 1544, and 1500 cm^−1^ appeared in the FT-IR spectrum of film (**A**) after the annealing process, which suggested the decomposition of the copper ions coordinated to compound **4** and the possibility of the formation of carbon sulfide. However, further investigations would be required to prove this hypothesis.

## 3. Experimental Section 

### 3.1. General

Chitosan (DAICHITOSAN 100D (VL), degree of deacetylation: 98%) (**1**) was kindly supplied by the Dainichiseika Color and Chemicals Manufacturing Company (Tokyo, Japan). *N*-Phenylthiocarbamoyl chitosan (**2**) with a DS_PhNHCS_ value of 0.85 was prepared from chitosan (**1**) according to a previously reported procedure [14]. All the other chemicals used in the study were purchased from commercial sources and used without further purification.

Fourier-transform-infrared (FT-IR) spectra were recorded on a Shimadzu IR Prestige-21 spectrophotometer (Shimadzu, Kyoto, Japan) as KBr pellets (1 mg of sample/200 mg of KBr). ^1^H and ^13^C NMR spectra were recorded on a Varian FT-NMR (500 MHz) spectrophotometer (Agilent Technologies, Santa Clara, CA, USA) using tetramethylsilane (TMS) as an internal reference standard in CDCl_3_. The standard number of scans for the ^1^H and ^13^C NMR measurements were 3500 and 22,000, respectively. Chemical shifts (δ) have been reported in parts per million (ppm). Gel permeation chromatography (GPC) was performed on a Shimadzu LC-10 system equipped with a Shimadzu UV-vis detector (SPD-10AVp) and a Shimadzu RI detector (RID-10A) under the following conditions: columns: K-802 + K-802.5 + K-805 in series; column temperature, 40 °C; flow rate, 1.0 mL/min; standards, polystyrene standards (Shodex, Tokyo, Japan). Thermogravimetric analysis (TGA) was conducted in nitrogen (flow rate: 60 mL/min) with a Shimadzu TGA-50 thermal analyzer. The samples for TGA were heated from 25 to 300 °C at a programming rate of 15 °C/min. UV-vis-NIR spectra were recorded on a Jasco V-560 UV-vis spectrophotometer (Jasco, Tokyo, Japan).

### 3.2. Preparation of Chitosan Derivative **4** and Copper Decanoate (**5**)

#### 3.2.1. *N*,*N*-(Decanoyl)phenylthiocarbamoyl Chitosan Decanoate (**4**)

Compound **2** (1.02 g, 3.38 mmol) and decanoic anhydride (11.0 g, 33.8 mmol) were added to pyridine (30 mL), and the resulting suspension was stirred at 60 °C for 24 h. The reaction was then cooled to ambient temperature and diluted with EtOAc. The organic layer was washed sequentially with a 1 M solution of aqueous HCl, a saturated aqueous solution of NaHCO_3_ and brine before being dried over anhydrous Na_2_SO_4_. The solvent was then removed under vacuum to afford crude products as an oil. The oil was added in a dropwise manner to EtOH (300 mL) with stirring to give a precipitate, which was collected by centrifugation (20,142× *g* 10 min) and washed twice with EtOH. A small portion of the isolated precipitate was dried under vacuum at ambient temperature for 6 h and used for the characterization of compound **3b** (0.682 g), whilst the remainder was used without drying for the preparation of compound **4**.

Aniline (3.1 mL, 33.8 mmol) was added to a solution of the wet precipitate in CHCl_3_ (10 mL), and the resulting mixture was stirred at 60 °C for 24 h. The reaction was then cooled to ambient temperature and poured into MeOH (100 mL) to give a precipitate, which was collected by centrifugation (15,000× *g*, 10 min) and dissolved in a small amount of EtOAc. The EtOAc solution was then added to MeOH (100 mL) in a dropwise manner to give a precipitate, which was collected by centrifugation (15,000× *g*, 10 min), washed with MeOH and dried under vacuum at ambient temperature for 6h to afford compound **4** as a white solid (1.43 g).

Compound **3b**: FT-IR (KBr) ν (cm^−1^): 3472, 3215, 2924, 2855, 2046, 1740, 1676, 1598, 1532, 1495, 1465, 1379, 1315, 1246, 1180, 1117, 1072, 752, 723, 698. ^1^H NMR (CDCl_3_): δ 12.1 (NH), 7.53–7.06 (phenyl-H), 5.17 (H-3), 4.48 (H-1, H-6a), 4.27 (H-6b), 3.68 (H-2, H-4, H-5), 2.35, 1.64, 1.27 (decanoyl –CH_2_–), 0.89 (decanoyl –CH_3_) ppm; ^13^C NMR (CDCl_3_): δ 186.3 (C=S), 178.0, 173.9, 173.3 (decanoyl C=O), 141.7 (NCS), 129.9, 129.0, 124.2 (phenyl-C), 103.1, 99.7 (C-1), 75.4 (C-4), 73.4 (C-5), 72.3 (C-3), 62.3 (C-6), 60.8, 59.5 (C-2), 34.0, 31.9, 29.3, 24.7, 22.7, (decanoyl –CH_2_–),14.1 (decanoyl –CH_3_) ppm.

Compound **4**: DS_phenylthiocarbamoyls_ = 0.82 (by elemental analysis); DPn = 78 (*M*w/*M*n = 2.56); FT-IR (KBr) ν (cm^−1^): 3474, 3339, 2926, 2855, 1740, 1676, 1599, 1537, 1499, 1462, 1377, 1319, 1246, 1163, 1112, 1063, 752, 725, 698. ^1^H NMR (CDCl_3_): δ 7.66–7.06 (phenyl-H), 5.31–3.48 (H-1,H-2, H-3, H-4, H-5, H-6a, H-6b), 2.35, 1.63, 1.26 (decanoyl –CH_2_–), 0.89 (decanoyl –CH_3_) ppm; ^13^C NMR (CDCl_3_): δ 179.3 (C=S), 173.3, 171.5 (decanoyl C=O), 136.5, 129.8, 124.2 (phenyl-C), 101.1 (C-1), 76.0-68.0 (C-3, C-4, C-5), 62.9 (C-6), 58.2 (C-2), 33.9, 31.9, 29.4, 24.7, 22.7, (decanoyl –CH_2_–),14.1 (decanoyl –CH_3_) ppm.

#### 3.2.2. Preparation of Copper(II) Decanoate (**5**)

Distilled water (3.5 mL) was added to a stirred solution of decanoic acid (1.40 g, 8.13 mmol) in petroleum ether (7 mL), followed by 30% aqueous ammonium (five drops by a disposal pipette) and a 1 M solution of copper sulfate (4.7 mL). The resulting mixture was then stirred at ambient temperature for 30 min before being extracted with CHCl_3_. The organic layer was then washed four times with distilled water, dried over Na_2_SO_4_ and concentrated under vacuum to give compound **5** as a blue solid.

Compound **5**: FT-IR ν (cm^−1^): 2920, 2851, 1597, 1582, 1467, 1433, 1416, 1315, 1186, 721, 669.

### 3.3. Preparation of a Cast Film from Compounds **4** and **5**

Cover glass (26 × 26 mm) was placed at the bottom of an aluminum foil cup with a diameter of 54 mm. A solution of compounds **4** in CHCl_3_ (8.16 × 10^−3^ mmol/mL, 10 mL) was then treated with a solution of compound **5** in the same solvent at a predetermined molar ratio. The resulting mixture was then stirred at ambient temperature for 15 min and poured into the aluminum foil cup. The cup was held at ambient temperature for 24 h and then heated at 200 °C for a predetermined period of time in an oven. The cup was then removed from the oven and cooled to ambient temperature to give cover glass with the resulting film.

## 4. Conclusions 

Target compound **3a** was not obtained under the conditions used in the current study because the isothiocyanation reaction proceeded during the decanoylation process. However, compound **4** with a high DS of phenylthiocarbamoyl groups (0.82) was obtained from this decanoylation process following an *N*-phenylthiocarbamoylation reaction. The cast film was prepared from a mixture of compound **4** and copper decanoate **5** in CHCl_3_ with a molar ratio of 1:5, and further annealed at 200 °C for 3 min to give a greenish film, which showed near-infrared absorption performance in the wide range of 800–2200 nm. This film could be easily prepared and be applied as a heat-ray shielding (or absorption) film and provides a further example of the high-value added utilization of chitosan derivatives.
